# Plasma cell-free DNA as predictor of disease status in patients with differentiated thyroid cancer - a prospective study from a tertiary care institution

**DOI:** 10.3389/fonc.2024.1473262

**Published:** 2024-10-24

**Authors:** Rashi Goel, Swayamjeet Satapathy, Kunal Ramesh Chandekar, Sanjana Ballal, Shipra Agarwal, Suryanarayan S. V. Deo, Madhavi Tripathi, Chandrasekhar Bal

**Affiliations:** ^1^ Department of Nuclear Medicine, All India Institute of Medical Sciences, New Delhi, India; ^2^ Department of Pathology, All India Institute of Medical Sciences, New Delhi, India; ^3^ Department of Surgical Oncology, All India Institute of Medical Sciences, New Delhi, India

**Keywords:** differentiated thyroid cancer, plasma, cell-free DNA, cfDNA, liquid biopsy, Qubit fluorometer

## Abstract

**Introduction:**

Plasma cell-free DNA (cfDNA) estimation offers a non-invasive method to potentially diagnose, monitor, and prognosticate patients with malignancy. This prospective study aimed to assess plasma cfDNA levels in patients with differentiated thyroid cancer (DTC) to determine its role in predicting disease status in the post-operative setting.

**Materials and methods:**

This was a single-center prospective observational study conducted at a public medical research university and hospital in New Delhi, India. 254 patients with DTC in the post-operative setting were included: 95 in Group 1 (active structural disease) and 159 in Group 2 (disease-free). Blood samples were collected for plasma separation and cfDNA extraction. The cfDNA concentrations were quantified and compared across various disease states.

**Results:**

Median values of plasma cfDNA (ng/µL) in groups 1 and 2 were found to be 0.272 (IQR: 0.137-0.442) and 0.222 (IQR: 0.123-0.398), respectively with no significant difference (p=0.122). cfDNA levels were significantly higher in patients in the age group ≥55 years (p=0.016). However, the cfDNA levels were not significantly associated with any of the other known prognostic markers of DTC.

**Discussion:**

Based on the results of this study, plasma cfDNA levels did not significantly predict disease status in patients with DTC in the post-operative setting.

## 1 Introduction

Thyroid cancer is the most prevalent endocrine malignancy, accounting for about 2% of all cancers (1, 2). Its diagnosis generally involves ultrasonography, fine-needle aspiration cytology (FNAC), conventional and scintigraphic imaging methods. Treatment for differentiated thyroid cancer (DTC) typically includes surgical removal of the thyroid gland followed by radioiodine therapy for patients at intermediate to high risk. Monitoring for potential recurrences or metastasis includes measuring serum thyroglobulin (Tg) levels, conventional CT/MR imaging, and [^131^I]I^-^ whole body scans (DxWBS) (3–6). In cases where DxWBS is negative but Tg levels are elevated, [^18^F]FDG PET/CT is particularly useful (7).

Approximately one-third of DTC patients develop radioactive iodine-refractory DTC (RR-DTC), which has a poor prognosis and requires early identification (8). However, there are still unresolved issues regarding the diagnostic definitions, therapeutic approaches, and follow-up strategies for thyroid cancers, indicating unmet medical needs.

Liquid biopsy, a non-invasive method, analyzes biological samples released from tumors such as circulating tumor cells (CTC), circulating tumor DNA (ctDNA) or even cell-free DNA (cfDNA) into body fluids. The potential advantages of liquid biopsy include minimal invasiveness, repeatability, and real-time monitoring of molecular changes, thus overcoming challenges associated with invasive tissue biopsies and imaging. It is presumed that cfDNA is released due to apoptosis, necrosis, degeneration of circulating tumor cells, and even from metastatic deposits (9–11) to the circulation, thus could be used as a suitable biomarker material for diagnosing of cancer. Recently, a few authors advocated that cfDNA monitoring can be successfully used for detection of tumors not visible on imaging and involves no radiation exposure, unlike radiological or nuclear imaging techniques (13–15). However, circulating tumor DNA constitute about 10% of cfDNA, the bulk of cfDNA comes from degeneration of white blood cells (12).

While cfDNA is found in various body fluids and contains genomic and mitochondrial DNA, the biological and pathological information it provides is often inconsistent and non-specific, raising questions about its reliability for diagnosis, prognosis estimation, and treatment response monitoring in oncology. The full nature and behavior of cfDNA are still not fully understood, casting doubt on its utility as a biomarker in oncology. Although it is promoted for detecting microsatellite instability, loss of heterozygosity, somatic mutations, polymorphisms, methylation, and integrity, the evidence supporting these claims is not robust (9, 13, 16). Furthermore, not all cfDNA alterations detected may be attributable to cancer, as increased levels of cfDNA can be found in benign diseases and tissue trauma (17, 18).

In the context of thyroid cancer, some studies report that cfDNA is useful in differentiating benign from malignant thyroid nodules and monitoring disease progression. However, their reliability is uncertain due to small sample sizes (19–21). In this large prospective study, we aimed to evaluate the potential role of plasma cfDNA in predicting disease status in post-operative DTC patients. We tested the null hypothesis that plasma cfDNA levels in post-operative DTC patients are similar between those who have active structural disease when compared to those who are disease-free/in remission (negative control). If the cfDNA values could separate out these two groups without overlapping, then cfDNA analysis could be validated as useful biomarker.

## 2 Materials and methods

### 2.1 Study design and patient population

This was a single-center prospective study conducted as a collaborative effort between the Departments of Nuclear Medicine, Pathology, and Surgical Oncology, at the All India Institute of Medical Sciences (AIIMS), New Delhi, India. The research protocol was approved by the Institute Ethics Committee (IECPG-158/24.03.2022). The study was conducted in accordance with the principles in the Helsinki Declaration and was carried out over a period of 20 months from the date of ethical clearance.

**Figure 1:**
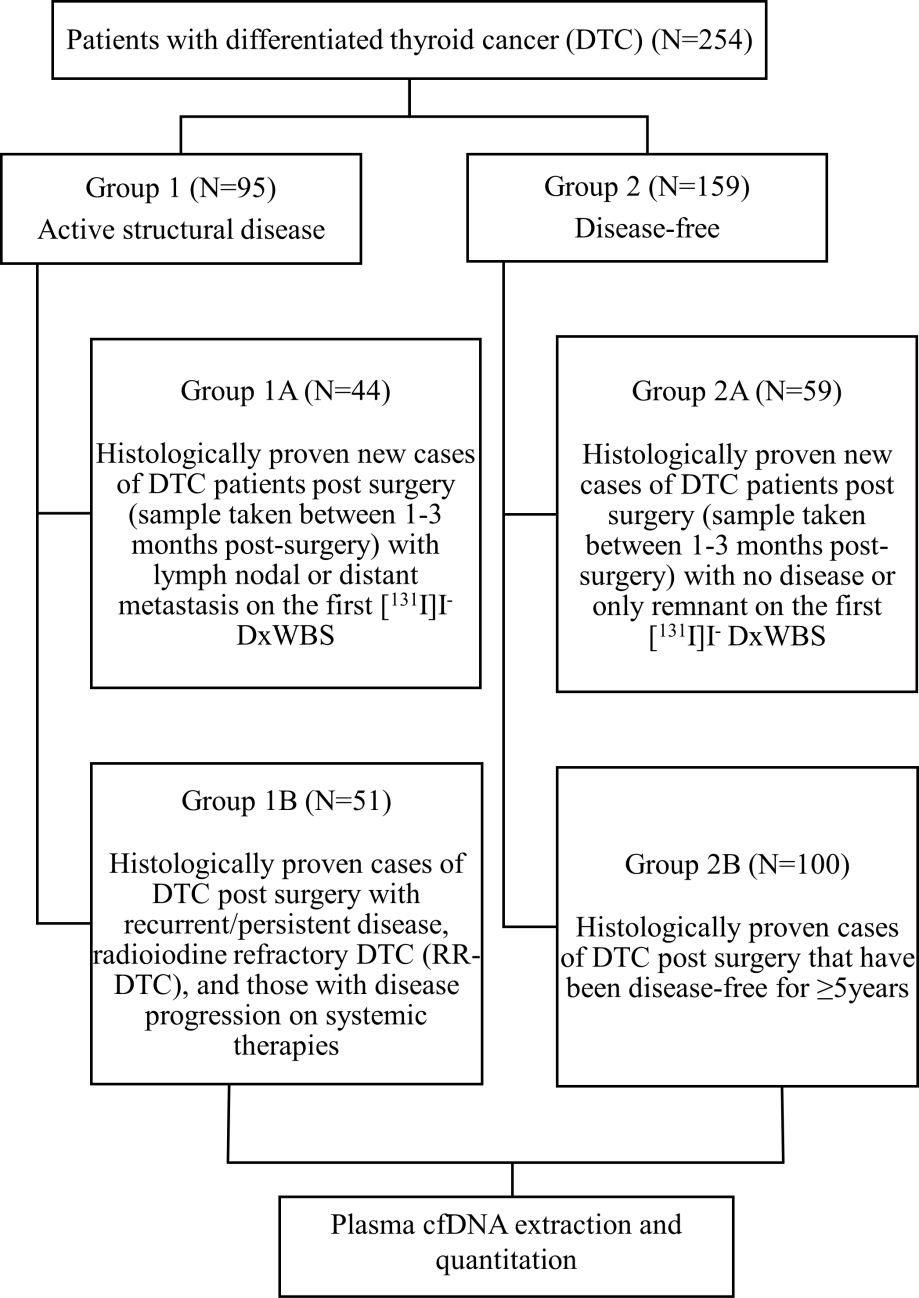
Flowchart illustrating study design.

Patients with histologically proven DTC (age ≥18 years) in the post-operative setting were recruited. The patients were recruited into two broad groups. Group 1 (active structural disease) included two subgroups - new cases of DTC post-surgery (blood sample taken between 1-3 months post-surgery) with lymph nodal or distant metastasis on the first [^131^I]I^-^ DxWBS (subgroup 1A) and patients with DTC post-surgery having recurrent/persistent disease, radioiodine refractory DTC (RR-DTC), and those with disease progression on systemic therapies (subgroup 1B). Group 2 (disease-free) also comprised of two subgroups - new cases of DTC post-surgery (blood sample taken between 1-3 months post-surgery) with no disease or only remnant tissue on first [^131^I]I^-^DxWBS (subgroup 2A) and patients with DTC post-surgery that have been disease-free for at least 5 years (subgroup 2B) (**Figure 1**). All the patients provided written informed consent. Patients suffering from any other malignancy and pregnant/lactating women were excluded from the study. The following details were recorded for each patient – age at diagnosis, sex, initial presenting complaint, surgical details, histopathology reports, biochemical investigations (serum Tg), [^131^I]I^-^ DxWBS, and their risk category was defined as per the 2015 American Thyroid Association (ATA) guidelines (4).

### 2.2 Blood sample processing and cfDNA extraction

10mL of patients’ blood was drawn from a peripheral vein and collected in ethylenediaminetetraacetic acid (EDTA) tubes. The tubes were inverted 8-10 times to mix the blood and anticoagulant and stored at 4 °C. The blood samples were centrifuged within 6-8 hours of being drawn at 3000g for 15 minutes at 4 °C. The supernatant plasma was carefully aspirated using a micropipette to not disrupt the cell layers and was transferred into 5mL Eppendorf tubes. The plasma was stored at -20 °C till further use. The plasma samples were thawed and equilibrated to room temperature before cfDNA extraction.

The cfDNA was extracted from 3mL plasma using the *ZYMO DNA Kit (Quick-cfDNA Serum & Plasma Kit Cat No: D-4076)* according to the instructions provided by the manufacturer. The cfDNA was eluted in 40µl elution buffer. The cfDNA concentration was then quantified using a Qubit fluorometer (Qubit™ 4 Fluorometer, Invitrogen by Thermo Fisher Scientific).

### 2.3 Statistical analyses

Statistical analyses were performed using IBM SPSS Statistics version 27.0. Sample size calculation was done using the G*Power software. Assuming power of 95% with two-sided alpha level of 5%, and sampling loss rate of 10%, a sample size of 254 patients was required to detect a mean difference of 0.1ng/µL between the diseased and non-diseased groups (allocation ratio ~ 1:1.5). Categorical data were expressed as frequencies and percentages. The normality of the continuous variables was checked by the Kolmogorov–Smirnov test. The non-normal continuous variables were expressed as median and interquartile range (IQR). The comparison of cfDNA among the two groups was made using the non-parametric Mann-Whitney U Test. The non-parametric Kruskal-Wallis Test (for > two groups) and Mann-Whitney U Test (for two groups) were used to determine the association between the levels of plasma cfDNA in DTC patients and other prognostic markers such as age, sex, pathological subtype, risk category, TNM stage, vascular invasion, capsular invasion, extra-thyroidal extension, focality of lesion and presence of distant metastasis. For comparisons between more than two groups, the significance values were adjusted by the Bonferroni correction as a *post-hoc* test. Results with two-sided p-value <0.05 were considered statistically significant.

## 3 Results

### 3.1 Demographic and clinical characteristics

A total of 254 patients with DTC were included in the study. Of these 254 patients, 95 belonged to Group 1 (active structural disease), and 159 belonged to Group 2 (disease-free). The demographic and clinical characteristics of the patients are summarized in **Table 1**. The median age of the entire cohort was 37 years (IQR:30-48 years), and a female preponderance (n=180/254, 70.8%) was noted. Of 254 patients, 220 (86.6%) had an initial presentation of a solitary thyroid nodule. Postoperatively, 223 of 254 (87.8%) patients had a histological diagnosis of papillary thyroid carcinoma (PTC). A majority (192/254, n=75.6%) of the patients had stage I disease and 113/254 (44.5%) of patients were classified as ATA low-risk. The demographic characteristics of the patients were observed to be comparable between the two groups. The groups 1 and 2 were further divided into subgroups 1A, 1B, 2A, and 2B as described in the Materials and Methods. The subgroup analysis of the demographic and clinical characteristics of the patients is summarized in **Table 2**.

**Table 1:** Demographic and clinical characteristics.

S.no	Parameter		GROUP 1 (Active structural disease) (N=95)	GROUP 2 (Disease-free) (N=159)
1	Age in years	Median (IQR)	45.0 (33.0-55.0)	35.0 (26.0-46.0)
		Range	18-77	18-73
2	Age category, n (%)	<55 years	67 (70.5)	145 (91.2)
		≥55 years	28 (29.5)	14 (8.8)
3	Sex, n (%)	Female	65 (68.4)	115 (72.3)
		Male	30 (31.6)	44 (27.7)
4	Initial Presentation, n (%)	Solitary thyroid nodule	78 (82.1)	142 (89.3)
		MNG	10 (10.5)	14 (8.8)
		Cervical lymph node(s)	2 (2.1)	3 (1.9)
		Metastases	5 (5.3)	0 (0.0)
5	Surgery, n (%)	TT/NTT/STT	89 (93.7)	136 (85.5)
		HT	6 (6.3)	23(14.5)
6	Nodal dissection, n (%)	Done	47 (49.5)	62 (39.0)
		Not done	48 (50.5)	97 (61.0)
7	Histopathology, n (%)	Papillary carcinoma	76 (80.0)	147 (92.4)
		Follicular carcinoma	15 (15.8)	10 (6.3)
		High-grade DTC	4 (4.2)	2 (1.3)
9	Pathological T stage (pT), n (%)	T1	13(13.7)	42 (26.4)
		T2	42 (44.2)	75 (47.2)
		T3	25 (26.3)	33 (20.8)
		T4	4 (4.2)	5 (3.1)
		Status unknown	11 (11.6)	4 (2.5)
10	Pathological N stage (pN), n (%)	N0	8 (8.4)	30 (18.9)
		N1	49 (51.6)	62 (39.0)
		NX	38 (40.0)	67 (42.1)
11	Focality, n (%)	Unifocal	81 (85.3)	144 (90.6)
		Multifocal	14 (14.7)	15 (9.4)
12	Stage, n (%)	I	43 (45.3)	149 (93.7)
		II	35(36.8)	9(5.7)
		III	1 (1.1)	1 (0.6)
		IVA	2 (2.1)	0 (0.0)
		1VB	14 (14.7)	0 (0.0)
13	Risk, n (%)	Low	2 (2.1)	111 (69.8)
		Intermediate	19 (20.0)	40 (25.2)
		High	74 (77.9)	8 (5.0)
14	Thyroglobulin (Tg)	Median (IQR)	69.5 (10.1-265.0)	0.2 (0.2-2.0)

MNG, multinodular goitre; TT, total thyroidectomy; STT, subtotal thyroidectomy; NTT, Near total thyroidectomy; HT, hemithyroidectomy; DTC, differentiated thyroid cancer.

**Table 2:** Demographic and clinical characteristics (sub-group).

S.no	Parameter		GROUP 1 (N=95)		GROUP 2 (N=159)	
			1A (N=44)	1B (N=51)	2A (N=59)	2B (N=100)
1	Age in years	Median (IQR)	34.5 (26.0-49.5)	51.0 (41.0-58.0)	34.0 (27.0-46.0)	35.0 (28.0-44.0)
		Range	18-77	24-75	19-73	18-65
2	Age category, n (%)	<55 years	37 (84.1)	30 (58.8)	50 (84.7)	95 (95.0)
		≥55 years	7 (15.9)	21 (41.2)	9 (15.3)	5 (5.0)
3	Sex, n (%)	Female	32 (72.7)	33 (64.7)	47 (79.7)	68 (68.0)
		Male	12 (27.3)	18 (35.3)	12 (20.3)	32 (32.0)
4	Initial Presentation, n (%)	Solitary thyroid nodule	33 (75.0)	45 (88.2)	51 (86.4)	91 (91.0)
		MNG	7 (15.9)	3 (5.9)	8 (13.6)	6 (6.0)
		Cervical lymph node(s)	1 (2.3)	1 (2.0)	0 (0.0)	3 (3.0)
		Metastases	3 (6.8)	2 (3.9)	0 (0.0)	0 (0.0)
5	Surgery, n (%)	TT/NTT/STT	43 (97.7)	46 (90.2)	54 (91.5)	82 (82.0)
		HT	1 (2.3)	5 (9.8)	5 (8.5)	18 (18.0)
6	Nodal dissection, n (%)	Done	21 (47.7)	26 (51.0)	29 (49.2)	33 (33.0)
		Not done	23 (52.3)	25 (49.0)	30 (50.8)	67 (67.0)
7	Histopathology, n (%)	Papillary carcinoma	35 (79.6)	41 (80.4)	54 (91.5)	93 (93.0)
		Follicular carcinoma	7 (15.9)	8 (15.7)	4 (6.8)	6 (6.0)
		High-grade DTC	2 (4.5)	2 (3.9)	1 (1.7)	1 (1.0)
9	Pathological T stage (pT), n (%)	T1	11 (25.0)	2 (3.9)	17 (28.8)	25 (25.0)
		T2	19 (43.2)	23 (45.1)	21 (35.6)	54 (54.0)
		T3	7 (15.9)	18 (35.3)	14 (23.7)	19 (19.0)
		T4	0 (0.0)	4 (7.8)	3(5.1)	2(2.0)
		Status unknown	7 (15.9)	4 (7.8)	4 (6.8)	0 (0.0)
10	Pathological N stage (pN), n (%)	N0	5 (11.4)	3 (5.8)	15 (25.4)	15 (15.0)
		N1	25 (56.8)	24 (47.1)	18 (30.5)	44 (44.0)
		NX	14 (31.8)	24 (47.1)	26 (44.1)	41 (41.0)
11	Focality, n (%)	Unifocal	36 (81.8)	45 (88.2)	52 (88.1)	92 (92.0)
		Multifocal	8 (18.2)	6 (11.8)	7 (11.9)	8 (8.0)
12	Stage, n (%)	I	26 (59.1)	17 (33.3)	57 (96.6)	92 (92.0)
		II	13 (29.5)	22 (43.1)	2 (3.4)	7 (7.0)
		III	0 (0.0)	1 (2.0)	0 (0.0)	1 (1.0)
		IVA	1 (2.3)	1 (2.0)	0 (0.0)	0 (0.0)
		1VB	4 (9.1)	10 (19.6)	0 (0.0)	0 (0.0)
13	Risk, n (%)	Low	2 (4.5)	0 (0.0)	29 (49.2)	82 (82.0)
		Intermediate	19 (43.2)	0 (0.0)	22 (37.3)	18 (18.0)
		High	23 (52.3)	51 (100)	8 (13.5)	0 (0.0)
14	Thyroglobulin in ng/mL	Median (IQR)	21.3 (1.3-208.7)	42.0 (1.0-388.0)	3.0 (2.0-17.0)	0.2 (0.2-0.2)

MNG, multinodular goitre; TT, total thyroidectomy; STT, subtotal thyroidectomy; NTT, Near total thyroidectomy; HT, hemithyroidectomy; DTC, differentiated thyroid cancer.

### 3.2 Comparison of plasma cfDNA levels between the two groups

**Figure 2:**
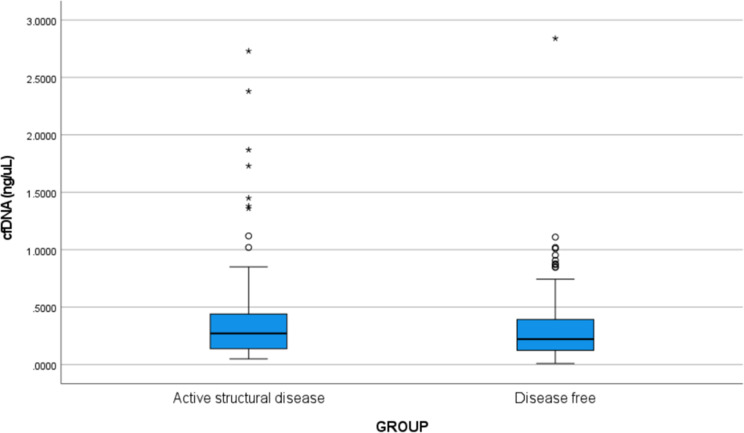
Box and whisker plot showing the cfDNA concentration (ng/µL) in Group 1 (active structural disease) and Group 2 (disease-free). The bold horizontal line splitting the boxes in two is the median. The lower and the upper sides of the box are the lower (Q1) and upper (Q3) quartiles. The median values of plasma cfDNA (ng/µL) in groups 1 and 2 were found to be 0.272 (IQR: 0.137-0.442) and 0.222 (IQR: 0.123-0.398) ng/µL, respectively. Slightly higher concentrations of cfDNA were observed in the patients with active structural disease, however, the difference was not statistically significant (p=0.122). Circles are outliers and asterisks are the extreme outliers.

The median values of plasma cfDNA (ng/µL) in groups 1 and 2were found to be 0.272 (IQR:0.137-0.442) and 0.222 (IQR:0.123-0.398) ng/µL, respectively. Slightly higher concentrations of cfDNA was observed in the patients with active structural disease, however, the difference was not found to be statistically significant (p=0.122). The box and whisker plot depicting the cfDNA concentration in the two groups is shown in **Figure 2**.

### 3.3 Subgroup analyses for plasma cfDNA levels

The median values of plasma cfDNA (ng/µL) in post-operative DTC patients with lymph node/distant metastasis on the first [^131^I]I^-^ DxWBS (subgroup 1A, n=44) and those with recurrent/persistent/progressive/RR-DTC (subgroup 1B, n=51) were 0.275 (IQR:0.122-0.505) and 0.272 (IQR:0.184-0.439), respectively. In contrast, the median values (ng/µL) in post-operative DTC patients with first [^131^I]I^-^ DxWBS showing no uptake outside the thyroid bed (subgroup 2A, n=59), and those disease-free for ≥5 years (subgroup 2B, n=100) were found to be 0.206 (IQR:0.101-0.290), and 0.248 (IQR:0.151-0.483), respectively. The differences in cfDNA levels between the subgroups at the time of first [^131^I]I^-^ DxWBS (subgroup 1A versus 2A), as well as those with long-term follow-up (subgroup 1B versus 2B), were not observed to be statistically significant (p=0.134, and p=1.000, respectively).

### 3.4 Plasma cfDNA levels and association with other prognostic markers in patients with DTC

We checked the association of cfDNA levels in patients with DTC with other prognostic markers such as patient age, sex, histopathology, TNM staging, multifocality of lesion, vascular invasion, capsular invasion, extra-thyroidal extension, pathological tumor (pT) and nodal (pN) stage of tumor, lymph node involvement, distant metastasis, and risk status. The results are summarized in **Table 3**. The cfDNA levels (ng/µL) were significantly higher in patients who were ≥55 years of age compared to those <55 years of age (median 0.324, IQR: 0.209-0.442 versus median 0.237, IQR: 0.120-0.419, p=0.016). However, the cfDNA levels were not significantly associated with any of the other known prognostic markers of DTC.

**Table 3:** Cell-free DNA levels and association with various other prognostic markers.

VARIABLES		Cell-free DNA concentration (ng/µL)	P value
		Median (IQR)	
Age	<55 years (N=212)	0.237 (0.120-0.419)	0.016
	≥55 years (N=42)	0.324 (0.209-0.442)	
Sex	Female (N=180)	0.231 (0.123-0.398)	0.149
	Male (N=74)	0.281 (0.159-0.478)	
Pathological subtype	Papillary carcinoma (N=223)	0.252 (0.129-0.412)	0.897
	Follicular carcinoma (N=25)	0.268 (0.116-0.460)	
	High-grade DTC (N=6)	0.274 (0.216-0.430)	
TNM staging	I (N=192)	0.245 (0.122-0.422)	0.103
	II (N=44)	0.220 (0.127-0.399)	
	III (N=2)	0.364 (0.195-0.363)	
	IVA (N=2)	0.297 (0.164-0.399)	
	IVB (N=14)	0.361 (0.271-0.761)	
Multifocality	Yes (N=29)	0.219 (0.096-0.348)	0.219
	No (N=225)	0.256 (0.131-0.430)	
Vascular invasion	Present (N=37)	0.191 (0.108-0.305)	0.06
	Absent (N=217)	0.268 (0.135-0.425)	
Capsular invasion	Present (N=14)	0.254 (0.111-0.456)	0.951
	Absent (N=240)	0.253 (0.129-0.416)	
Extra-thyroidal extension	Present (N=19)	0.222 (0.123-0.292)	0.319
	Absent (N=235)	0.257 (0.128-0.427)	
Pathological T stage, pT*	T1 (N=55)	0.184 (0.108-0.303)	0.022
	T2 (N=117)	0.281 (0.160-0.448)	
	T3 (N=58)	0.258 (0.136-0.459)	
	T4 (N=9)	0.260 (0.219-0.334)	
Pathological N stage, pN**	Present (N=111)	0.223 (0.117-0.389)	0.384
	Absent (N=38)	0.242 (0.146-0.519)	
Distant metastases	None (N=205)	0.246 (0.123-0.410)	0.452
	Lungs (N=22)	0.275 (0.154-0.513)	
	Bones (N=27)	0.272 (0.165-0.444)	
RISK	Low (N=113)	0.250 (0.138-0.423)	0.510
	Intermediate (N=59)	0.188 (0.120-0.433)	
	High (N=82)	0.269 (0.158-0.419)	

* Status unknown in 15 patients.

** Status unknown in 105 patients.

## 4 Discussion

Estimating plasma cfDNA has been proposed as a promising non-invasive method for diagnosing, monitoring, and prognosticating cancer patients, though the supporting evidence remains weak (22, 23). Recent studies have examined the role of cfDNA in patients with thyroid nodules and thyroid cancer (21, 24). However, to our knowledge, no study has comprehensively assessed and compared plasma cfDNA levels in post-surgical DTC patients across different disease states. This study aims to fill this gap by quantitatively evaluating plasma cfDNA levels in post-surgical DTC patients and comparing them across various disease states to determine its clinical utility in predicting disease status.

We measured cfDNA levels using a Qubit fluorometer (QubitTM 4 fluorometer, Invitrogen by Thermo Fisher Scientific) due to its ease of use, efficiency, and comparable measurements to quantitative polymerase chain reaction (qPCR) (25). Our analysis included plasma cfDNA levels from 254 DTC patients, categorized into groups with active structural disease and those without (disease-free). The study was adequately powered to detect a minimum difference of 0.1ng/µL in cfDNA levels between the two groups based on previous smaller studies (21, 22). However, our results showed only a minimal, non-significant difference of 0.05ng/µL in median plasma cfDNA levels between the active structural disease group and the disease-free group. Additionally, no significant difference in plasma cfDNA concentration was found between patients with recurrent/persistent/progressive disease and those who remained disease-free on long-term follow-up.

Previous studies have reported a significant decrease in cfDNA concentrations following surgery in cancer patients, indicating the primary tumor as the major source of preoperatively raised cfDNA (10, 21). However, our study, focusing on post-operative patients, did not observe meaningful differences in cfDNA levels across different disease states, suggesting that perhaps only the primary tumor significantly contributes to plasma cfDNA levels. This needs to be re-validated in multicentric larger sample studies. Another possible explanation for the similarity in cfDNA levels across groups could be the low-grade nature of DTCs, which may release smaller amounts of circulating cfDNA compared to more aggressive cancer types (26). No significant difference has been demonstrated between different histotypes of DTC such as papillary and follicular sub-types (27).

Most of our disease-free patients had Stage I disease, characterized by low disease burden, which may have contributed to reduced tumor DNA shedding into the bloodstream, but patients with extensive structural disease also showed lower cfDNA is puzzling observation. Thus, the lack clear separation of cfDNA values between positive and negative controls puts the question mark on the utility of cfDNA as a valid biomarker in patients with DTC.

There are other limitations of liquid biopsies that may restrict their widespread use. The small quantity of cfDNA in the blood can make detection and sequencing challenging and costly. Achieving standardization across different laboratories and vendors is crucial for consistent results. Additionally, not all cfDNA alterations detected may be attributable to cancer, as bulk of increased levels of cfDNA can be found in benign lesions, autoimmune diseases, inflammatory diseases, and tissue trauma (17, 18). These limitations may be partially offset by estimating ctDNA levels as it is derived specifically from tumor cells, containing the same genetic mutations and alterations as the primary tumor. This specificity allows for more accurate identification and monitoring of cancer-related changes compared to cfDNA, which includes DNA from both tumor and normal cells (12, 28). Plasma ctDNA also serves as an alternative to tumor tissues for detecting mutations and companion diagnostic purposes. The analysis of the clinical utility of ctDNA for cancer care includes qualitative and quantitative assessment (29). Sato et al. (2021) studied the role of plasma ctDNA carrying the BRAFV600E mutation in 22 PTC patients before and after surgery in predicting outcomes, finding that detection of the BRAFV600E mutation in presurgery plasma can provide information on the local progression of the primary tumor and the presence of mutated BRAFV600E in postsurgery ctDNA might predict PTC recurrence (30). However, its routine clinical utility is still limited by its low sensitivity, technical complexity, and lack of standardization (28, 29). CtDNA levels have also been shown to correlate with metastatic status (31). However, studies regarding role of plasma cfDNA levels in metastatic thyroid cancer are lacking.

Contradictory data exist regarding the association of cfDNA in DTC patients with various clinicopathological features of thyroid cancer. While some studies report higher cfDNA levels associated with high-risk features of malignancy, others find no significant relation between cfDNA concentrations and clinicopathological features of thyroid cancer (21, 32).

Dutta et al. (2021) analyzed the relationship between plasma cfDNA with histopathological parameters of thyroid cancer in 37 patients with DTC and found that significantly higher cfDNA levels were associated with high-risk features of malignancy such as nodal involvement, vascular & capsular invasion, extrathyroidal extension, and advanced tumor stage (21). Conversely, Klimaite et al. (2022) estimated the concentration of plasma cfDNA using qPCR in 68 patients with PTC and found no statistically significant relation between cfDNA concentrations and various clinicopathological features of PTC such as age, sex, pathological tumor stage, lymph node metastases, pathological subtype, extrathyroidal extension, and lymphovascular invasion (32). In our study, cfDNA levels were significantly higher (p=0.016) in patients in the age group ≥55 years, aligning with the known adverse prognosis for this age group. However, the cfDNA levels were not significantly associated with any of the other known prognostic markers of DTC such as sex, pathological subtype, TNM staging, lymph nodal involvement, vascular invasion, capsular invasion, extra-thyroidal extension, primary tumor focality, risk category.

Overall, our study suggests caution in relying solely on plasma cfDNA levels for predicting disease status in DTC patients. A multimodal approach, combining clinical, imaging, and molecular information, may provide a more accurate assessment of disease status. Although our initial hypothesis regarding cfDNA as a predictor for disease status in DTC patients was not supported by our data, this negative result provides valuable insights for refining future research directions and underscores the need for a comprehensive approach to biomarker discovery and validation in thyroid cancer.

Our study has limitations, including the lack of specific mutation detection from cfDNA samples, single-time-point blood sample collection, and exclusion of pre-operative DTC patients. Nonetheless, the prospective nature of our study, adequate power, and inclusion of patients at different disease spectra are significant strengths.

## 5 Conclusion

In conclusion, our study found that plasma cfDNA levels did not significantly predict disease status in post-operative patients with DTC. Apart from age, plasma cfDNA levels also did not show an association with other prognostic markers of malignancy, casting doubt on its relevance as a biomarker for DTC. Overall, these findings raise concerns about the reliability of plasma cfDNA levels in predicting disease status in DTC patients.

## Data Availability

De-identified patient data can be made available to bonafide researchers affiliated to an institution upon reasonable request to the corresponding author following publication.
